# Taking the Next Step: How Can Implementation Science Advance Diabetes Foot Care for Rural and Remote Australians?

**DOI:** 10.1002/jfa2.70156

**Published:** 2026-05-04

**Authors:** Angela Byrnes, James Charles, Susanna Cramb, Sarah Jensen, Trent Johnston, Sarah Larkins, Steven McPhail, Jaap J. van Netten, Christina N. Parker, Ruth Tulleners, Zephanie Tyack, Jonathan Golledge, Peter A. Lazzarini, QFeet Study Group

**Affiliations:** ^1^ Australian Centre for Health Services Innovation and Centre for Healthcare Translation School of Public Health and Social Work Queensland University of Technology Brisbane Australia; ^2^ School of Health Sciences and Social Work Griffith University Brisbane Australia; ^3^ Department of Podiatry Metro North Hospital and Health Service Brisbane Australia; ^4^ Department of Podiatry Townsville Hospital and Health Service Townsville Australia; ^5^ Anton Breinl Research Centre James Cook University Townsville Australia; ^6^ Department of Rehabilitation Medicine Amsterdam Movement Sciences Amsterdam UMC University of Amsterdam Amsterdam the Netherlands; ^7^ School of Nursing Queensland University of Technology Brisbane Australia; ^8^ Queensland Research Centre for Peripheral Vascular Disease College of Medicine and Dentistry James Cook University Townsville Australia; ^9^ Department of Vascular and Endovascular Surgery Townsville University Hospital Townsville Australia; ^10^ Allied Health Research Collaborative Metro North Hospital and Health Service Brisbane Australia

**Keywords:** diabetes‐related foot disease, implementation science, multidisciplinary care, rural and remote health, telehealth

## Abstract

Diabetes‐related foot disease (DFD) is a leading cause of disability worldwide. In Australia, DFD affects approximately half a million people and is the primary driver of diabetes‐related hospitalisations, amputations and costs. Guideline‐based multidisciplinary footcare can halve these rates and improve quality of life, yet access remains inequitable, particularly for rural and remote communities for whom DFD hospitalisation and amputation rates are persistently high. Geographic isolation, workforce shortages and fragmented service delivery are barriers to DFD care, with Aboriginal and Torres Strait Islander Peoples experiencing additional cultural and systemic challenges. Telehealth‐enabled models of care offer a promising solution to reducing inequities in access without compromising effectiveness. Four ‘Foot Hubs’ have been established across Queensland (Australia) to deliver specialist multidisciplinary footcare via a hub‐and‐spoke model, combining telehealth, outreach, and local partnerships to improve access for people living with DFD in rural and remote areas. This commentary provides an introductory overview of these Foot Hub services and how implementation science (the scientific study of methods and strategies to promote the systematic and sustainable uptake of new practices) can support the uptake and sustainability of these new models of care.

## 1 Background

Globally, diabetes‐related foot disease (DFD) ranks 13th in disease burden, surpassing better‐known conditions like dementia and breast cancer [1, 2, 3]. This DFD burden is driven predominantly by disability, rather than premature mortality, related to reduced quality of life and increased rates of hospitalisation and amputation [1, 2, 3]. As the population ages and disability becomes a greater component of overall disease burden, the World Health Organisation has called on nations to prioritise research that focuses on discovering effective interventions to reduce the global disability burden [2].

In Australia, DFD affects an estimated half a million people and is the leading cause of diabetes‐related hospitalisations, amputations and reduced quality of life, particularly affecting those experiencing greater socioeconomic disadvantage [4, 5, 6, 7]. Multidisciplinary footcare that aligns with international and national guidelines in Australia [4, 8, 9] has been shown to halve amputation [10, 11] and hospitalisation rates [12] while remaining cost‐effective even after accounting for higher care costs [13]. Despite this, and consistent with global trends, people living in rural and remote areas of Australia have persistently higher rates of DFD hospitalisation and amputation compared to metropolitan areas [4, 12]. This commentary reviews healthcare access disparities for people with DFD living in rural/remote areas, explores telehealth's potential to improve access through new models of care and considers how implementation science can support their uptake.

### 1.1 Disparities in Healthcare for People With DFD Living in Rural and Remote Areas of Australia

Australia is a geographically dispersed nation, with over a quarter of its population living in rural/remote areas [14]. Rural/remote communities have higher rates of modifiable health risk factors, poorer access to healthcare services, and in turn poorer health outcomes compared to people living in metropolitan areas [14]. Furthermore, rural/remote areas of Australia can have higher proportions of individuals with complex healthcare needs, including older people, those living with disability, and/or Aboriginal and Torres Strait Islander Peoples [14].

Disparities in access to and quality of healthcare for people living in rural/remote areas stem from a lack of healthcare infrastructure, challenges attracting and retaining skilled healthcare professionals and poor service coordination [15, 16]. Subsequently, people living in rural/remote areas face lengthy travel, high accommodation costs and fragmented care, impacting on chronic disease outcomes in particular [16]. Additional barriers to care are experienced by Aboriginal and Torres Strait Islander peoples, who are a culturally diverse group and encounter inefficiency in the healthcare system [17, 18]. Despite great resilience, Aboriginal and Torres Strait Islander peoples are still facing the impacts of colonisation and dispossession, often not recognised by healthcare systems [17, 18]. The removal and dislocation from community and lack of culturally sensitive care negatively impact the health and wellbeing of Aboriginal and Torres Strait Islander peoples [15, 16, 17, 18].

Qualitative research with healthcare providers in rural/remote areas across Australia identified access to specialist DFD care, poor care integration and inadequate footcare as major gaps in DFD services for rural/remote communities [19]. Indeed, less than half of people with DFD in major cities receive guideline‐based footcare [13], and these rates are much lower for people living in rural/remote areas [4, 20]. These factors have likely contributed to those living in rural/remote areas having significantly worse DFD outcomes compared with those living in a major city, including ulcer healing, being ulcer‐free and amputation outcomes [4, 20, 21, 22]. Given that timely provision of guideline‐based care from multidisciplinary footcare teams has been shown to be effective and cost‐effective for people with DFD [11, 13, 20], a major challenge to reducing the large global DFD burden lies in providing equitable care to rural/remote communities around the world [23].

## 2 New Models of Healthcare for People With DFD Living in Rural and Remote Areas

Virtual care, such as telehealth, has been proposed as a promising solution to increase access to specialist care for people living in rural/remote areas and has seen a rapid increase in acceptability and use since the COVID‐19 pandemic [24]. The use of virtual care in facilitating access to specialist DFD care is not new [25, 26], with trials conducted in Australia and overseas showing that care delivered by multidisciplinary footcare teams achieves comparable DFD outcomes when delivered predominantly via telehealth or in‐person [27, 28, 29].

**FIGURE 1:**
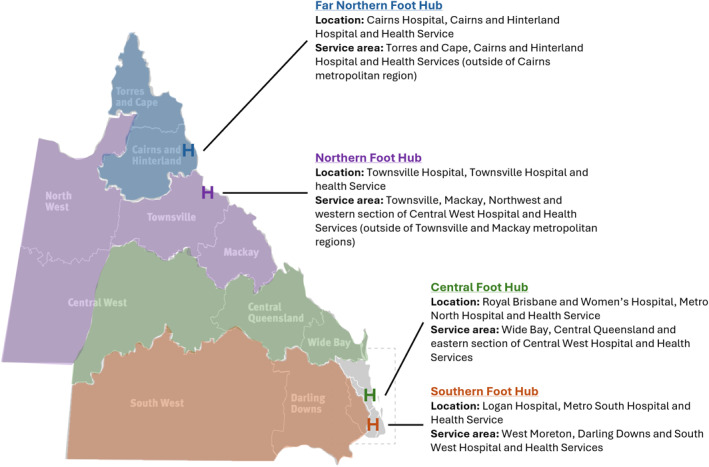
Foot Hub locations and their service areas.

**FIGURE 2:**
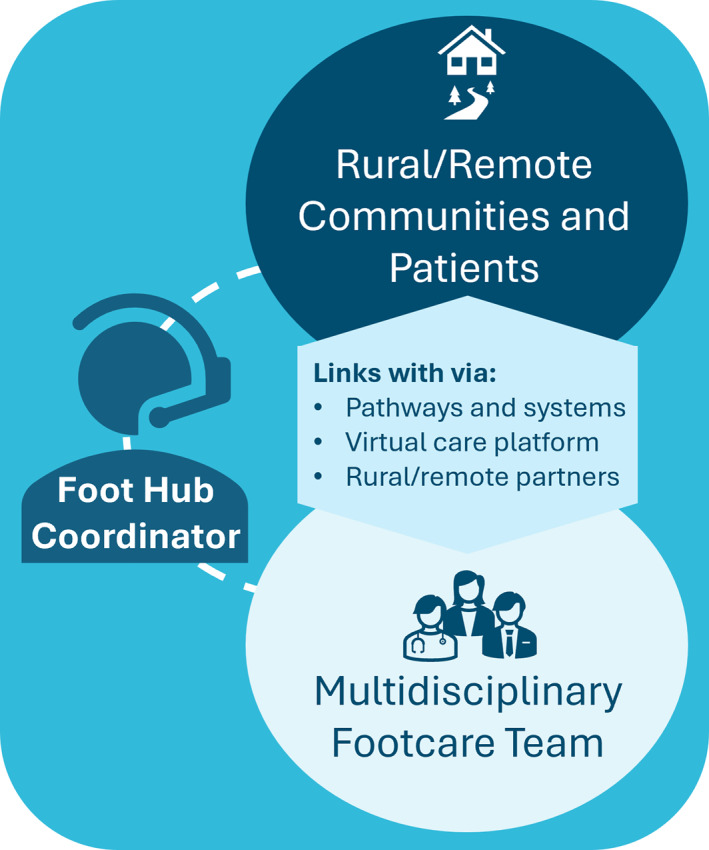
Foot Hub service components.

In 2023, to help address the persistently high DFD hospitalisation and amputation rates in rural/remote areas [4, 30], the Queensland Government in Australia committed ∼AU$5 million annually in recurrent funding to establish four novel telehealth‐based multidisciplinary footcare teams (termed ‘Foot Hubs’) [31]. Each Foot Hub is based in a city with a tertiary hospital (selected due to existing vascular surgery escalation pathways) and services people with DFD in allocated areas of Queensland, providing coverage to all rural/remote areas within the state (Figure 1). Using a hub‐and‐spoke model, the Foot Hubs have three central components: (i) a dedicated multidisciplinary footcare team of physicians (e.g., endocrinologist and infectious disease consultant), surgeons (e.g., vascular surgeon and orthopaedic surgeon), nurses (e.g., wound care nurse and nurse practitioner), allied health (e.g., podiatrist, diabetes educator, dietitian, psychology and social worker), administrative support and access to Aboriginal and Torres Strait Islander healthcare workers; (ii) a Foot Hub Coordinator, typically a senior podiatrist, that is, the conduit between the multidisciplinary footcare team and spoke site healthcare providers and patients; and (iii) an online platform of telehealth infrastructure, clinical pathways, referral and data collection systems that help connect patients in rural/remote area spoke sites with the Foot Hub (Figure 2). These Foot Hubs endeavour to partner with healthcare providers and facilities in rural/remote areas, including hospitals, community healthcare centres, primary healthcare centres, Aboriginal and Torres Strait Islander community‐controlled health organisations and general practices to deliver care closer to home for persons with DFD. A key feature of the Foot Hub model is the flexible, hybrid delivery of services that include telehealth, multidisciplinary case conferencing, outreach clinics and face‐to‐face (outpatient/inpatient) clinics.

Early data on the uptake of the Foot Hubs suggest they have generally increased access to dedicated multidisciplinary footcare teams for people with DFD residing in rural/remote areas. However, early reflections from one service highlighted ongoing barriers and enablers, and identified further mixed‐methods studies are required to determine the (cost‐) effectiveness of these services [32].

## 3 Using Implementation Science to Enhance Care and Outcomes

Implementing complex interventions containing several interacting components across multiple system levels is challenging in the dynamic healthcare setting and poor implementation can result in missed health benefits and an inability to sustain change [33, 34]. Success of these Foot Hubs is therefore contingent on their implementation and uptake into routine practice, particularly the engagement of spoke site healthcare providers. Implementation science applies methods and strategies to promote the systematic and sustainable uptake of new practices [35] and is underpinned by theories, models and frameworks (collectively termed ‘theoretical approaches’) that can be used to plan, guide and understand success or failure of specific implementation projects [36]. These theoretical approaches can be broadly categorised as guiding the implementation process (process frameworks), describing underlying determinants (determinant frameworks) and/or providing a scaffolding for evaluation (evaluation frameworks). An example of each is provided in Table 1.

**TABLE 1:** Summary of example implementation frameworks within each category.

Name of framework	Category	Phase of implementation	Brief description
EPIS [37]	Process (and determinant)	Planning/designing, implementing, evaluating	Derived from socio‐ecological (e.g., diffusion of innovations), organisational (e.g., values‐innovation fit) and facilitation theories, EPIS also draws on systems thinking.
			As a process framework, EPIS outlines four key phases of the implementation process, identifying factors that play a key role during each phase. These phases are nonlinear and dynamic and include:
			1. Exploration: Decision made whether to adopt an innovation by considering existing or emergent clinical or public health needs and available innovations to address those needs.
			2. Preparation: Preparing for implementation by identifying contextual factors, adapting innovations to fit implementation contexts and developing an implementation plan that includes implementation supports.
			3. Implementation: Innovation use is initiated and instantiated, guided by the implementation plan and supports previously identified, with ongoing monitoring and adaptation as necessary.
			4. Sustainment: Structures, processes and supports are established and ongoing, allowing continued delivery of the innovation with ongoing adaptations as necessary.
			EPIS highlights the role of *bridging factors*, which are the structures and processes that connect the inner and outer contexts.
i‐PARIHS [38]	Determinant (and process)	Planning/designing, implementing, evaluating	The i‐PARIHS framework is underpinned by theories of innovation, behavioural and organisational change and improvement (e.g., diffusion of innovations, theory of organisational readiness for change and normalisation process theory) as well as theoretical perspectives on facilitation as an enabling approach.
			As a determinant framework, i‐PARIHS describes factors at multiple levels that promote (enablers) or hinder (barriers) successful implementation. The framework outlines four core constructs:
			1. Characteristics of the innovation (the ‘thing’ to be implemented)
			2. Recipients (individuals and teams affected by and influencing implementation)
			3. Context (local, organisational and outer settings)
			4. Facilitation.
			These factors can be explored to better explain or predict implementation success and understand complexities. Facilitation in the i‐PARIHS framework is framed as being the core process through which the innovation is implemented into the given context.
RE‐AIM [39, 40]	Evaluation	Planning/designing, evaluating	High level framework that supports the evaluation of particularly public health interventions to understand impact to support decision‐making regarding ongoing investment. RE‐AIM draws on theories of diffusion of innovations, ecological models and evaluation frameworks that emphasise both individual and organisational levels of change.
			RE‐AIM includes five dimensions that assess the impact and sustainability of interventions:
			1. Reach: Proportion and representativeness of individuals who receive an intervention.
			2. Effectiveness: Impact on important outcomes, including potential negative effects.
			3. Adoption: Proportion and representativeness of settings and staff that adopt the intervention.
			4. Implementation: Fidelity to, adaptations and cost of delivery of the intervention.
			5. Maintenance: Long‐term sustainability at both individual and organisational levels.
			RE‐AIM was developed for use in evaluating multilevel (e.g., individual, clinic or organisation and community) interventions that incorporate multiple components (i.e. policy, environmental and individual) and is intended to be used for real‐world application.

Abbreviations: EPIS, Exploration Preparation Implementation Sustainment; i‐PARIHS, Integrated Promoting Action on Research Implementation in Health Services; RE‐AIM, Reach Effectiveness Adoption Implementation Maintenance.

**FIGURE 3:**
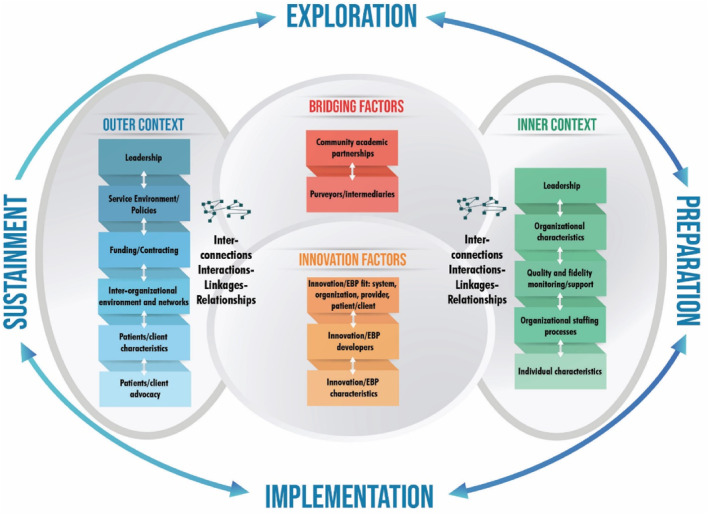
Exploration, Preparation, Implementation and Sustainment (EPIS) framework. The above figure was originally published in the paper by Moullin et al. [37] and has been reproduced without changes under the Creative Commons Attribution 4.0 International License (http://creativecommons.org/licenses/by/4.0/).

Implementation science approaches are gaining traction in health service practice and research [41]; however, these approaches are infrequently used in DFD research and practice [42]. The ‘QFeet Study’ is a new multi‐site study employing an implementation science approach to support ongoing implementation and sustainability of the Foot Hubs through the application of theory informed frameworks (e.g., the Exploration, Preparation, Implementation and Sustainment (EPIS) framework [37], see Figure 3 and description in Table 1). The EPIS framework was selected as it provides structured guidancefactors (i.e., determinants) to consider across all phases of the implementation process and explicitly acknowledges the dynamic interplay between internal organisational and external system contexts. This includes inter‐organisational relationships, which are expected to be influential when implementing across diverse contexts within the large geographical areas served by each Foot Hub. The framework will be used to guide stakeholder engagement (informal and formal through qualitative interviews), community consultation (e.g., ‘town halls’) and align implementation strategies with identified determinants to develop implementation and sustainability plans. These Foot Hub services will also be robustly evaluated using a hybrid Type II effectiveness‐implementation study design (which includes a prospective cohort and retrospective administrative data linkage studies, as well as multi‐method qualitative studies), with implementation and evaluation outcomes aligned with the RE‐AIM framework [43] (see Table 1). Future programmes may benefit from reporting of implementation processes and outcomes through the identification of effective strategies, which will also inform scaling of these services in new contexts.

## 4 Conclusion

Significant gaps exist in delivering guideline‐based care for people with DFD, which disproportionately affects individuals residing in regional/remote areas. Virtual, coordinated, specialist multidisciplinary footcare in the form of novel Foot Hubs provided largely via telehealth is a promising new model for increasing access to timely, high quality footcare for people living with DFD in rural/remote areas. Taking the next step into implementation science should support the increased uptake of Foot Hub models with spoke sites serving rural/remote communities, enhancing impact and sustainability, while generating evidence of (cost‐)effectiveness to enable future scale and spread of these innovative models.

## Author Contributions

**Angela Byrnes:** conceptualization, writing – original draft, writing – review and editing. **James Charles:** conceptualization, funding acquisition, writing – review and editing. **Susanna Cramb:** conceptualization, funding acquisition, writing – review and editing. **Sarah Jensen:** conceptualization, funding acquisition, writing – review and editing. **Trent Johnston:** conceptualization, funding acquisition, writing – review and editing. **Sarah Larkins:** conceptualization, funding acquisition, writing – review and editing. **Steven McPhail:** conceptualization, funding acquisition, writing – review and editing. **Jaap J. van Netten:** conceptualization, funding acquisition, writing – review and editing. **Christina N. Parker:** conceptualization, funding acquisition, writing – review and editing. **Ruth Tulleners:** conceptualization, writing – review and editing. **Zephanie Tyack:** conceptualization, funding acquisition, writing – review and editing. **Jonathan Golledge:** conceptualization, funding acquisition, writing – review and editing. **Peter A. Lazzarini:** conceptualization, funding acquisition, writing – review and editing.

## Funding

The QFeet study has been funded by a National Health and Medical Research Council (NHMRC) Partnership Grant (#2032912), led by A/Prof Peter Lazzarrini. Research by Jonathan Golledge is supported by grants from the Australian National Health and Medical Research Council (GNT2041176/GNT2026319/GNT1180736), Medical Research Futures Fund (MRF2032898/MRF2022807/MRF2015979/MRF2015817/MRF2015999), Queensland Government (SCRF), Heart Foundation and Townsville Hospital and Health Services.

## Conflicts of Interest

The authors declare no conflicts of interest.

## Permission to Reproduce Material From Other Sources

The Exploration, Preparation, Implementation and Sustainment (EPIS) Framework figure (presented as Figure 3) was originally published in an Open Access paper by Moullin et al. [37] and has been reproduced without changes under the Creative Commons Attribution 4.0 International License (http://creativecommons.org/licenses/by/4.0/).

## Data Availability

Data sharing not applicable to this article as no datasets were generated or analysed during the current study.
